# Entry closure using the Najuta stent graft without supra-aortic vessel revascularization of a chronic type B aortic dissection with an aberrant right subclavian artery

**DOI:** 10.1016/j.jvscit.2025.101850

**Published:** 2025-05-20

**Authors:** Sho Takagi, Yoshihiro Goto, Junji Yanagisawa, Yui Ogihara, Yasuhide Okawa

**Affiliations:** Department of Cardiovascular Surgery, Toyohashi Heart Center, Toyohashi, Japan

**Keywords:** Aberrant subclavian artery, Chronic dissection, Endovascular repair, Entry closure, Najuta

## Abstract

We report a case of entry closure using the Najuta fenestrated stent graft without supra-aortic vessel revascularization in a patient with chronic type B aortic dissection and an aberrant right subclavian artery. The Najuta fenestrated stent graft was inserted and deployed at zone 0. Complete preservation of the neck vessels and entry closure were achieved. This approach is a less invasive yet effective option for treating chronic type B aortic dissection with an aberrant subclavian artery.

Entry closure by thoracic endovascular aortic repair (TEVAR) can be effective for aortic aneurysms with dissection in the late chronic stage after false lumen enlargement. Creating a proximal landing zone in the intact aorta may require bypasses to the supra-aortic vessels, which are more invasive than simple TEVAR. TEVAR for aortic arch lesions, especially those with anomalies, can be complex. The Najuta fenestrated stent graft (NFSG) is a patient-specific device that is preshaped and includes fenestrations. These fenestrations are not reinforced with any structural support. According to the manufacturer's instructions, the fenestration openings are designed to be larger than the ostia of the supra-aortic branches; therefore, placing a bare metal or covered stent in these branches is not always required. Iwakoshi et al^1^ reported that TEVAR with the NFSG could result in favorable patency rates for all branches. Additionally, Isernia et al^2^ reported that the NFSG is safe and effective for cases involving high surgical risks. We report a case of entry closure using the NFSG without supra-aortic vessel revascularization in a patient with chronic Stanford type B aortic dissection (TBAD) and an aberrant right subclavian artery (ARSA). The patient agreed to the publication of this report after receiving sufficient information, and institutional approval was obtained.

## Case report

**Fig 1:**
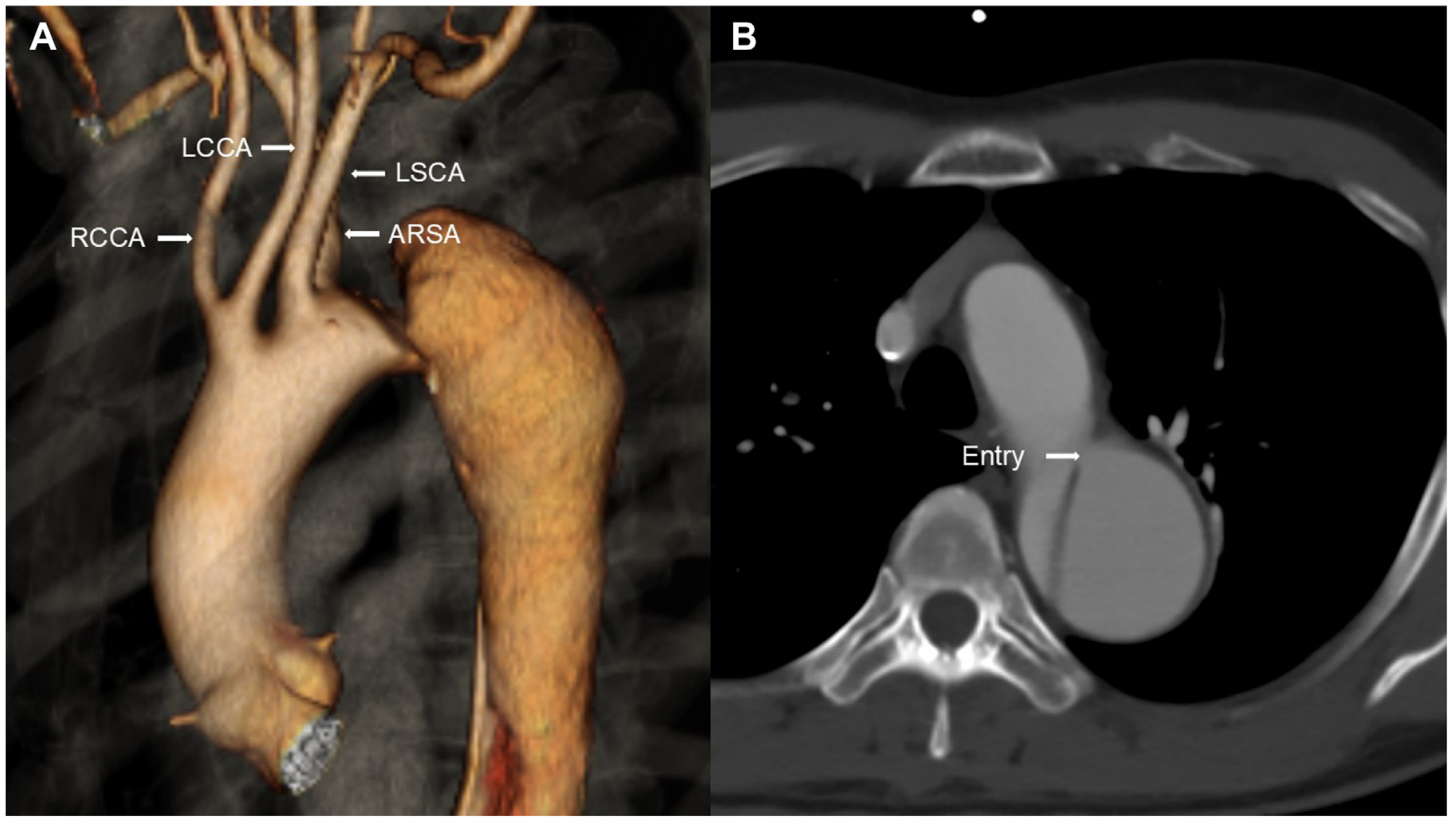
Preoperative contrast-enhanced computed tomography (CT) images. **(A** and **B)** CT imaging with contrast revealed Stanford type B aortic dissection (TBAD) with an entry tear in zone 3 with an aberrant right subclavian artery (ARSA). CT scan showed that the entry of the aortic dissection was located below the ARSA. The sequence of the cervical branches was as follows: a common origin of the right common carotid artery (RCCA) and left common carotid artery (LCCA); left subclavian artery (LSCA); and ARSA. The ARSA originated from the aortic arch.

**Fig 2:**
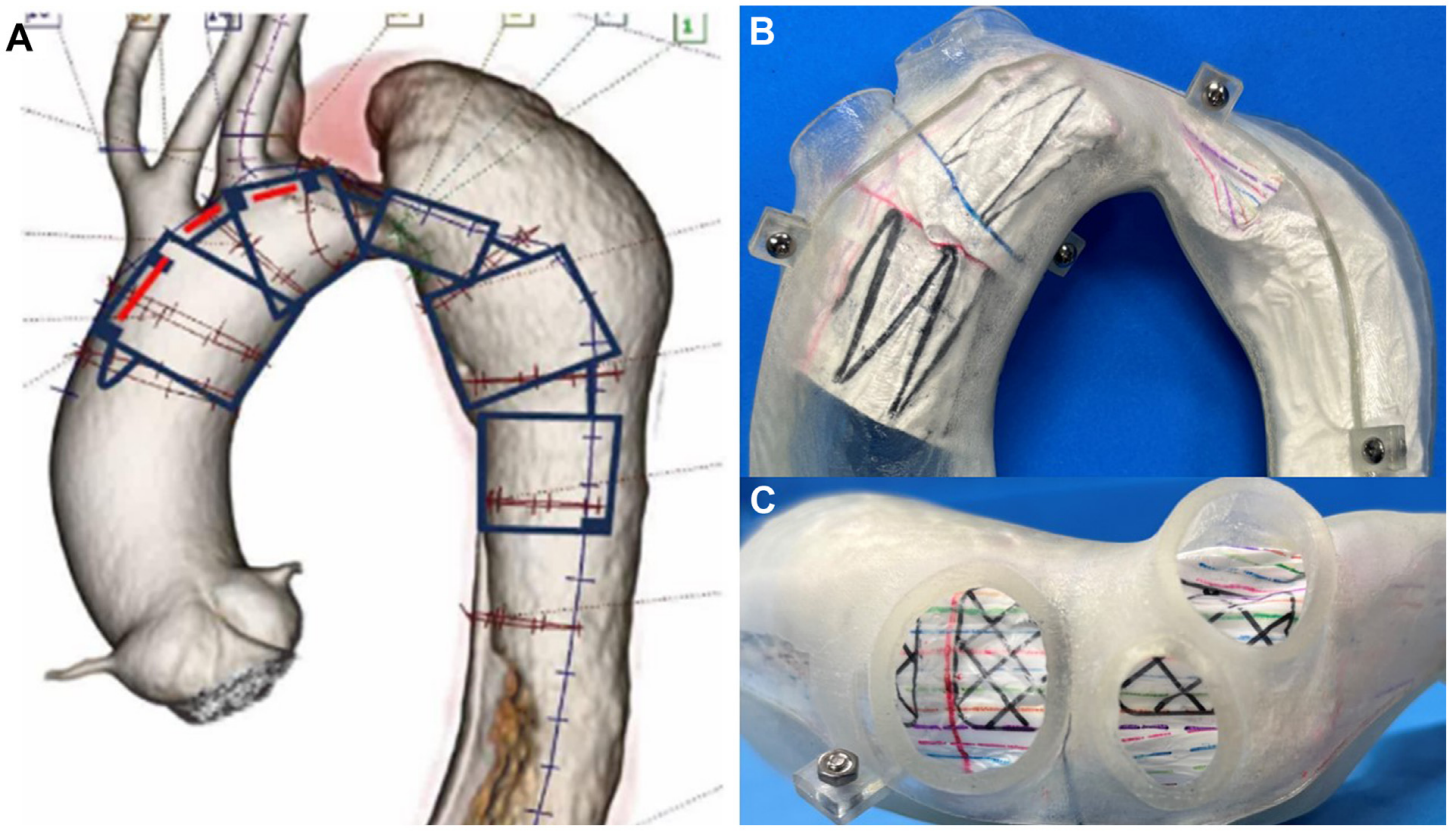
Device implantation plan, customized full-scale stent graft model, and plaster model. **(A)** The planning diagram for the deployment of the Najuta in zone 0 based on three-dimensional (3D) computed tomography (CT) scans. The red lines indicate the fenestration sites. **(B** and **C)** The customized stent graft model in the plaster model. The mesh-lined areas represent the fenestration sites.

A 54-year-old man with a history of hypertension and pneumothorax was referred to our hospital for treatment of a thoracic aortic aneurysm. Computed tomography (CT) imaging with contrast revealed TBAD with an entry tear in zone 3 and an ARSA (Fig 1, *A* and *B*). CT scans revealed that the aortic dissection entry was located 10 mm below the ARSA (Fig 1, *B*). The sequence of the cervical branches was as follows: a common origin of the right common carotid artery and left common carotid artery (LCCA), left subclavian artery (LSCA), and ARSA. The ARSA originated from the aortic arch (Fig 1, *A*). The diameter of the thoracic aortic aneurysm that was enlarged by chronic aortic dissection was 54 mm. The chronic aortic dissection extended distally to the bilateral iliac arteries, and the superior mesenteric artery (SMA) and left renal artery were involved. The patient did not experience acute aortic dissection. Although the onset of dissection was unknown, chronic dissection was diagnosed based on the CT findings. To control the enlargement of the false lumen, we planned entry closure using TEVAR. Because the entry tear was close to the origins of the LSCA and ARSA, creating a proximal sealing zone in the intact aorta without dissection required bypasses to the LSCA and ARSA. Because less invasive treatment was preferred, we planned TEVAR using the NFSG system in zone 0 (Fig 2, *A*-*C*). The NFSG is a custom-made device that requires 3 to 4 weeks for manufacturing; therefore, production was initiated based on the patient-specific three-dimensional (3D) CT imaging results.^3^^,^^4^

**Fig 3:**
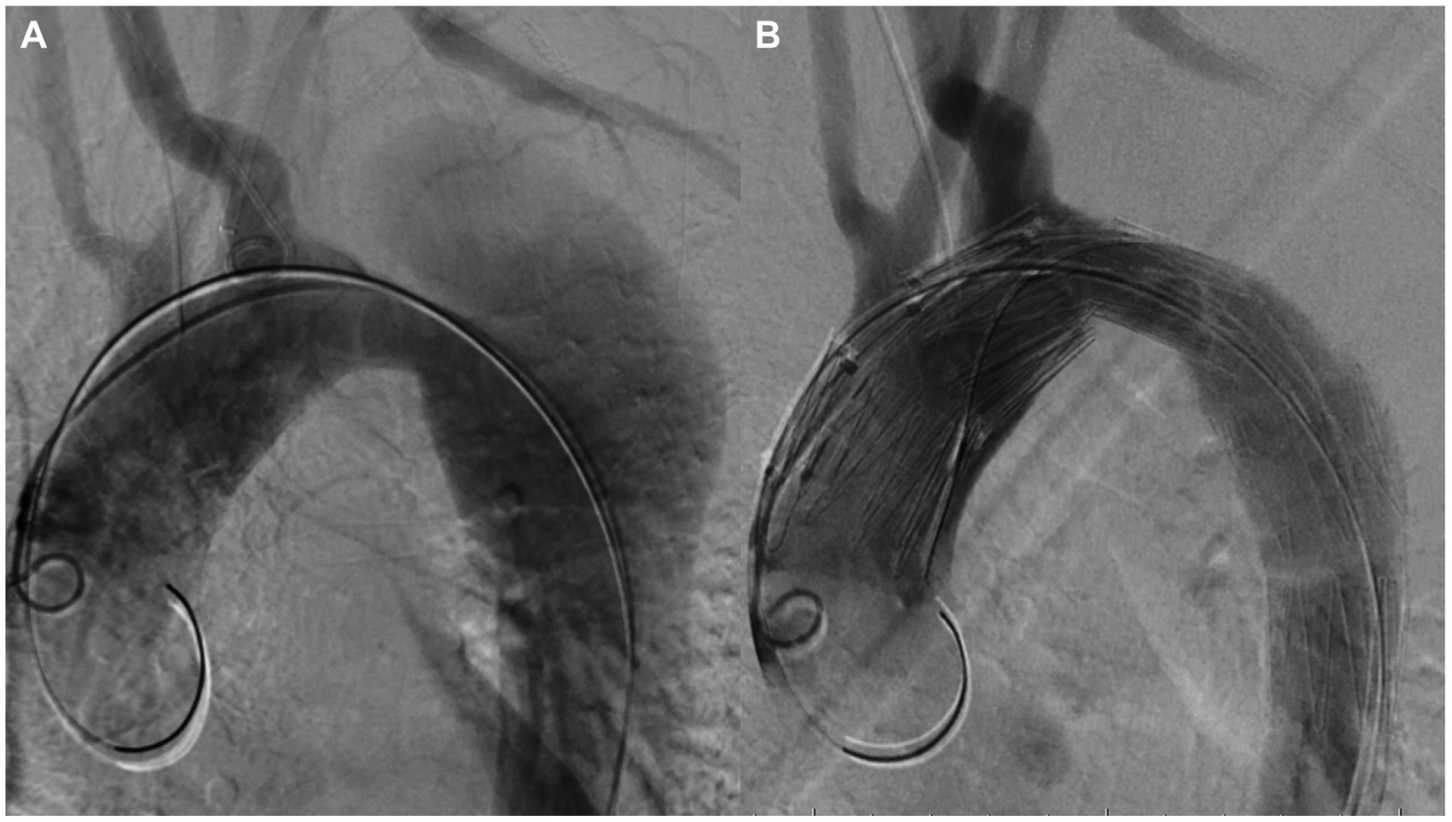
Intraoperative angiography findings. **(A)** Intraoperative angiography revealed a dissecting aneurysm with an entry tear in zone 3 and an aberrant right subclavian artery (ARSA). **(B)** Completion angiography demonstrated complete entry closure and preserved flow into the four neck vessels.

**Fig 4:**
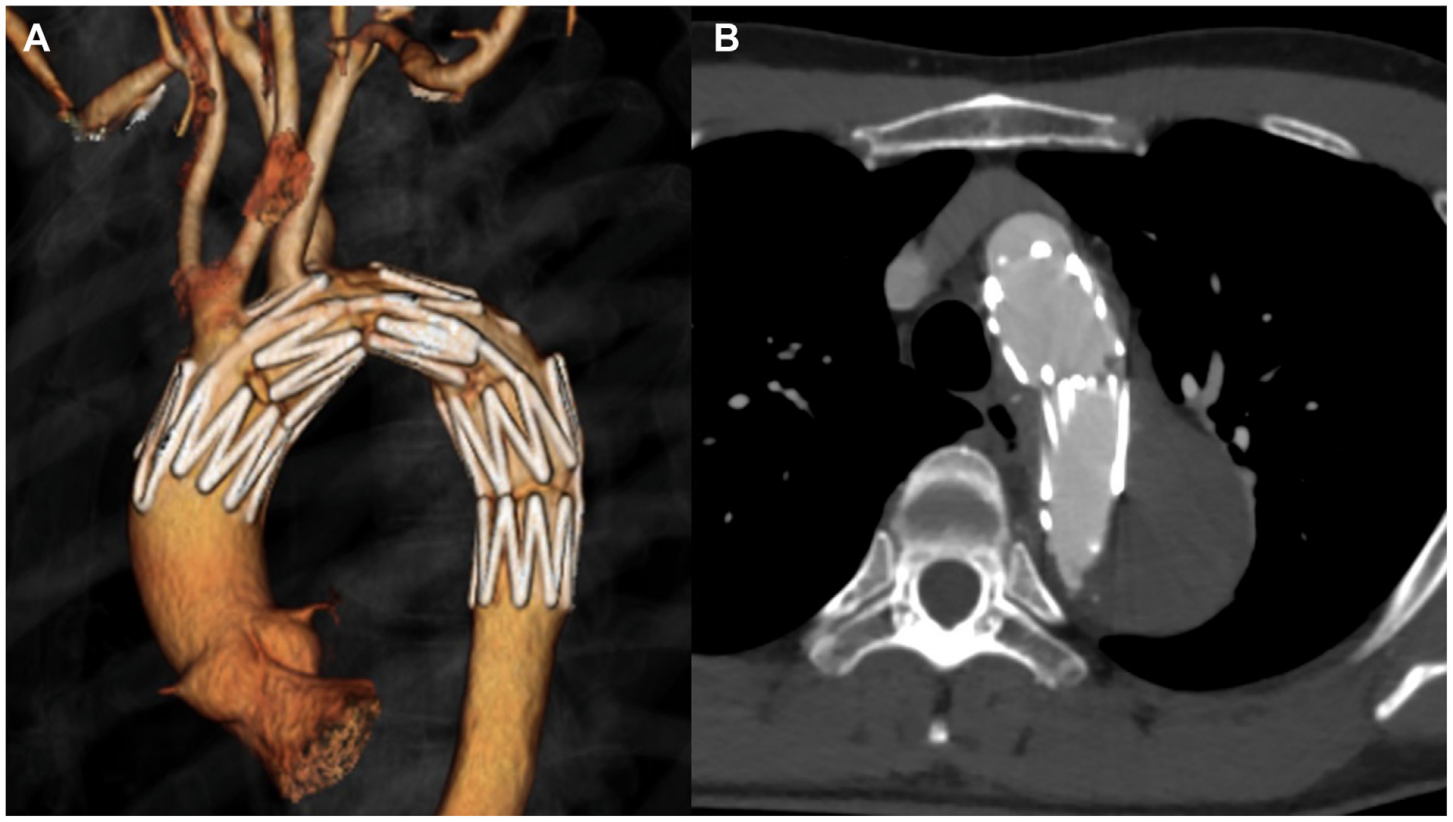
Postoperative contrast-enhanced computed tomography (CT) images. **(A** and **B)** Postoperative contrast-enhanced CT revealed entry closure and patency of the four neck vessels.

During general anesthesia, percutaneous vascular access was obtained on the right common femoral artery (CFA) and both brachial arteries. An 8F sheath was placed in the right CFA. A 6F double-lumen introducer sheath, which is a vascular access sheath with a 6F outer diameter and two independent internal lumens, was introduced from the left brachial artery. Another 6F sheath was inserted via the right brachial artery. A 4F pigtail catheter was positioned in the ARSA as a marker during deployment of the NFSG. Intraoperative angiography revealed a dissecting aneurysm with an entry tear in zone 3 and an ARSA (Fig 3, *A*). The 8F sheath was replaced with a 24F sheath using a Lunderquist wire (Cook Medical, Inc., Bloomington, IN). A 0.032-inch Radifocus wire (Terumo, Tokyo, Japan) was introduced in place of the Lunderquist wire and guided from the left brachial artery to the right CFA. The 38-mm NFSG, which is a customized device tailored based on patient-specific 3D CT imaging results,^3^^,^^4^ was introduced using a 23F U-shaped sheath with continuous tension applied by traction at both ends of the wire. Because of the steep curvature of the aortic arch, the NFSG could not be delivered to zone 0 using the Radifocus wire. Therefore, the Radifocus wire was replaced with a Lunderquist wire, which was transported and deployed to the proximal sealing zone of zone 0. Digital subtraction angiography was conducted before and after NFSG deployment to verify accurate alignment and maintain perfusion to the supra-aortic vessels. Before surgery, precise realignment to the positioning of the fenestrations of the NFSG was performed using an anatomically accurate plaster model produced by a 3D printer^5^ (Fig 2, *B* and *C*) to ensure proper alignment with the anatomical points of the cervical arteries. Completion angiography demonstrated complete entry closure and preserved flow into the four neck vessels (Fig 3, *B*). A postoperative contrast-enhanced CT scan revealed entry closure and patency of the four neck vessels (Fig 4, *A* and *B*). The patient was discharged 3 days after TEVAR. He seemed energetic during his follow-up evaluation 6 months after discharge, and aortic events and occlusion of the four neck vessels were not observed. Additionally, false lumen thrombosis from the aortic arch to the level of the SMA was observed, and aortic remodeling seemed to progress from the dissecting aneurysm to above the SMA.

## Discussion

TEVAR can be effective for aortic aneurysms with dissection in the late chronic stage after false lumen enlargement, and it can result in favorable immediate and mid-range results.^6^ Although follow-up procedures, such as reentry closure because of upward blood flow,^7^^,^^8^ are often necessary after endovascular management of chronic TBAD, the primary objectives of TEVAR are to control false lumen expansion, mitigate rupture risk, and maintain aortic integrity. With late-stage chronic TBAD, the false lumen often becomes rigid and less compliant compared with the false lumen observed with acute cases. However, because of advancements in endovascular devices, TEVAR has become significantly easier, even for chronic TBAD cases. In the present case, TEVAR using the NFSG was effective and safe for our patient with chronic TBAD and an ARSA associated with a dissecting aortic aneurysm. In this case, the entry was close to the origins of the LSCA and ARSA. Traditional approaches may have required bypasses to both subclavian arteries, which are invasive; however, using the NFSG enabled a less invasive procedure.

The NFSG is a tailor-made device consisting of a self-expanding stainless-steel Z-stent combined with an expanded polytetrafluoroethylene (ePTFE) graft.^3^^,^^4^ To customize the NFSG, a patient-specific 3D aortic arch model was generated using CT angiography data. Before TEVAR, a full-scale customized stent graft model was deployed onto the plaster model (Fig 2, *A* and *B*). Several studies have reported that TEVAR using the NFSG is associated with a low incidence of aorta-related events, high patency rates of the supra-aortic vessels,^1^^,^^9^ and safe deployment of the NFSG.^2^ This approach enabled precise placement of the NFSG and may help to prevent migration without requiring neck vessel reconstruction.^5^^,^^10^ The fenestrated design of the NFSG facilitates the creation of a long proximal sealing zone; therefore, TEVAR is a viable option for treating anomalous aortic arches, as demonstrated in this case. In some instances, TEVAR may necessitate subclavian artery revascularization to achieve an adequate proximal sealing length. Traditionally, subclavian artery revascularization has been performed surgically, for example, using a LCCA-to-LSCA bypass or LSCA-to-LCCA transposition. Recently, a variety of endovascular interventions, including chimney grafts, branched endografts, and fenestrated grafts such as the NFSG, have been introduced for subclavian artery revascularization during TEVAR.^11^

Because the Najuta stent is housed within a graft, its radial force is relatively low.^5^ However, this characteristic prevents new stent graft-induced entry during endovascular treatment of TBAD. The ePTFE graft and Najuta stents are sutured only at the top and bottom. This means that the ePTFE graft and Najuta stents are secured solely at the proximal and distal ends of the system, thus creating a graft expansion effect; therefore, the graft is highly suitable for sealing entry tears. The NFSG features an internal skeleton and is designed to gently seal the entry tear with its graft expansion effect; therefore, it may be beneficial for treating aortic dissection. As described, the NFSG has features that make it advantageous for treating aortic dissection. However, it is a custom-made device that requires 3 to 4 weeks for production; therefore, it is unsuitable for cases involving acute aortic dissection.

## Conclusions

Our case provides insights regarding the entry closure of chronic TBAD. Entry closure using the NFSG without supra-aortic vessel revascularization is a less invasive and effective option for patients with anomalous aortic arches like an aberrant subclavian artery.

## Funding

None.

## Disclosures

None.
